# Mimotopes and Proteome Analyses Using Human Genomic and cDNA Epitope Phage Display

**DOI:** 10.1002/cfg.174

**Published:** 2002-06

**Authors:** Mullaney P. B., Marks D. J., Pallavicini G. M.

**Affiliations:** 1 Departments of Laboratory Medicine (BPM, MGP) and Radiation Oncology (MGP) Anesthesia and Pharmaceutical Chemistry (JDM), and Cancer Center University of California at San Francisco San Francisco CA 94143 USA; 2 Box 0808 UCSF Cancer Center 2340 Sutter St Rm 429 UCSF San Francisco CA 94115-0808 USA

## Abstract

In the post-genomic era, validation of candidate gene targets frequently requires proteinbased strategies. Phage display is a powerful tool to define protein-protein interactions by generating peptide binders against target antigens. Epitope phage display libraries have the potential to enrich coding exon sequences from human genomic loci. We evaluated genomic and cDNA phage display strategies to identify genes in the 5q31 Interleukin gene cluster and to enrich cell surface receptor tyrosine kinase genes from a breast cancer cDNA library. A genomic display library containing 2 × $10^{6}$ clones with exon-sized inserts was selected with antibodies specific for human Interleukin-4 (IL-4) and Interleukin-13. The library was enriched significantly after two selection rounds and DNA sequencing revealed unique clones. One clone matched a cognate IL-4 epitope; however, the majority of clone insert sequences corresponded to *E. coli* genomic DNA. These bacterial sequences act as ‘mimotopes’ (mimetic sequences of the true epitope), correspond to open reading frames, generate displayed peptides, and compete for binding during phage selection. The specificity of these mimotopes for IL-4 was confirmed by competition ELISA. Other *E. coli* mimotopes were generated using additional antibodies. Mimotopes for a receptor tyrosine kinase gene were also selected using a breast cancer SKBR-3 cDNA phage display library, screened against an anti-erbB2 monoclonal antibody. Identification of mimotopes in genomic and cDNA phage libraries is essential for phage display-based protein validation assays and two-hybrid phage approaches that examine protein-protein interactions. The predominance of *E. coli* mimotopes suggests that the *E. coli* genome may be useful to generate peptide diversity biased towards protein coding sequences.

Abbreviations used: IL, interleukin; ELISA, enzyme linked immunoabsorbant assay; PBS, phospho-buffered saline; cfu, colony forming units.


**Comparative and Functional Genomics**
*Comp Funct Genom* 2002; **3**: 254–263.
Published online 10 May 2002 in Wiley InterScience (www.interscience.wiley.com). **DOI:** 10.1002/cfg.174

**Research Article**

### Mimotopes and proteome analyses using human genomic and cDNA epitope phage display

B. P. Mullaney, J. D. Marks and M. G. Pallavicini*
*Departments of Laboratory Medicine (BPM, MGP) and Radiation Oncology (MGP), Anesthesia and Pharmaceutical Chemistry (JDM), and Cancer Center, University of California at San Francisco, San Francisco, CA 94143, USA*

**Correspondence to:*
*Box 0808, UCSF Cancer Center, 2340 Sutter St, Rm 429 UCSF, San Francisco, CA 94115-0808, USA.*
*E-mail: palla@cc.ucsf.edu*

Received: 4 October 2001
Accepted: 22 April 2002

### Abstract

**In the post-genomic era, validation of candidate gene targets frequently requires protein-based strategies. Phage display is a powerful tool to define protein-protein interactions by generating peptide binders against target antigens. Epitope phage display libraries have the potential to enrich coding exon sequences from human genomic loci. We evaluated genomic and cDNA phage display strategies to identify genes in the 5q31 Interleukin gene cluster and to enrich cell surface receptor tyrosine kinase genes from a breast cancer cDNA library. A genomic display library containing $2 \times 10^6$ clones with exon-sized inserts was selected with antibodies specific for human Interleukin-4 (IL-4) and Interleukin-13. The library was enriched significantly after two selection rounds and DNA sequencing revealed unique clones. One clone matched a cognate IL-4 epitope; however, the majority of clone insert sequences corresponded to *E. coli* genomic DNA. These bacterial sequences act as 'mimotopes' (mimetic sequences of the true epitope), correspond to open reading frames, generate displayed peptides, and compete for binding during phage selection. The specificity of these mimotopes for IL-4 was confirmed by competition ELISA. Other *E. coli* mimotopes were generated using additional antibodies. Mimotopes for a receptor tyrosine kinase gene were also selected using a breast cancer SKBR-3 cDNA phage display library, screened against an anti-erbB2 monoclonal antibody. Identification of mimotopes in genomic and cDNA phage libraries is essential for phage display-based protein validation assays and two-hybrid phage approaches that examine protein-protein interactions. The predominance of *E. coli* mimotopes suggests that the *E. coli* genome may be useful to generate peptide diversity biased towards protein coding sequences.**

**Abbreviations used: IL, interleukin; ELISA, enzyme linked immunoabsorbant assay; PBS, phospho-buffered saline; cfu, colony forming units.** **Copyright © 2002 John Wiley & Sons, Ltd.**

**Keywords:** **phage display; genomic libraries; proteomics; epitope mapping; mimotopes**

### Introduction

A major challenge of the post-genomic era is to link DNA sequence with encoded proteins. Functional characterization of genes identified by human genome sequencing requires exploration of protein-protein interactions. Protein-protein interactions are governed partially by the sequence and conformation of interacting peptides; thus, strategies to identify and characterize sequence-specific protein-protein interactions are an important component of functional genomics and proteomics. Phage display libraries facilitate investigation of the molecular basis of protein-protein interactions [18]. For example, phage display peptide libraries [24] have been used to characterize antibody-epitope interactions [2,7,9] and phage display cDNA libraries have been used to define protein-protein interactions involving Hepatitis C, kinase, or monoclonal antibody epitopes [5,13,20,22,27]. Epitope and antibody libraries [17] facilitate functional genomic analyses because members that link genomic sequence with protein can be selected.

Identification of coding regions is a key step in

Copyright © 2002 John Wiley & Sons, Ltd.

linking genome sequence with expressed proteins. Computational analysis of DNA sequence has been used extensively to predict coding regions [1,25]. Protein-based methodologies that enrich coding (exon) sequences from non-coding (intron) sequences would be complementary to computational approaches by facilitating linkage of genotype with protein phenotype. Genome-protein linkage is particularly relevant for diseases, such as cancer, where genomic alterations (i.e., amplification, deletion, translocation, etc.) are prevalent, yet the spectrum of expressed genes encoded and expressed by these altered regions is often unknown. Phage approaches have been used to display small genomes, such as Hepatitis C virus [20,22] or prokaryotic artificial chromosomes [10,14,15], but to our knowledge, there are no examples of phage displayed human genomic sequences.

We evaluated a phage display strategy to identify coding exon sequences from defined regions of the genome. In this approach, epitope phage display libraries from specific regions of the human genome were enriched for coding exon sequences that bind to target proteins (i.e., antibodies). The approach was designed to maximize library diversity and the likelihood of exon display, and to minimize sequence space devoted to introns and stop codons. The intron-exon pattern of gene structure dictates that epitopes generated from genomic fragments will encode primarily linear, small exon-specific epitopes. For example, *in silico* sequence analyses of the 5q31 Interleukin gene region indicate that the majority of the exons within this region range between 100–300 bp (**http://www.lbnl.gov/lifesciences**). Variables related to genomic sequence, such as size of the target region (kilobase, megabase, etc.), gene location within six reading frames, stop codon frequency and in-frame sequences are important considerations in developing phage display-based coding exon identification. In addition, proper cloning orientation is required for successful pIII phage display. An insert sequence must be in-frame relative to the leader sequence and continue in-frame into the pIII sequence [3]. A stop codon within the insert sequence will cause a premature truncation of the peptide and prevent surface display. We used size selection strategies to optimize exon display from genomic fragments, derived from a 50 kb human P1 artificial chromosome, containing genes from the 5q31 Interleukin gene cluster. An epitope library from breast cancer cell cDNA was also was evaluated for gene specific protein-protein interactions. The success and challenges of creating and screening epitope display libraries from large genomic fragments and cDNAs are discussed.

### Materials and methods

### Genomic epitope library construction and characterization

The H11 library was constructed from a 50 kb human P1 (P1 clone 876h9, Genbank accession AC004039), containing the Interleukin-4, Interleukin-13, and kinesin-like protein-3 genes from 5q31. 20 μg P1 DNA was purified by a standard method (Qiagen) [6] and was randomly fragmented with decreasing concentrations of DNase I (10 units/ml) in 10 mM Tris pH 7.0/10 mM $MnCl_2$ for 8 minutes at 15°C, extracted and precipitated. Fragments were blunted with 5 units/μg T4 polymerase for 30 min at 12°C, extracted and precipitated. Linkers containing a *Sfi*I restriction site (Link1 5′-AGCGGCCG CAGGCCATGGAGGCC, Link2 5′-GGCCTCCA TGGCCTGCGGCCGCT) were ligated to target DNA with 400 units T4 DNA ligase for 2 hours at room temperature. The resulting product was electrophoresed on a 2.0% agarose gel and the size range of 100–300 bp was collected and eluted from NA-45 DEAE paper (Schleicher and Schuell, Keene, NH). 100 ng of the linker-ligated product was used as template in PCR with a nested primer LP5 (5′-GCGGCCGCAGGCCATGGA) with 5.0 units *Pfu* Polymerase (Stratagene, La Jolla, CA) for 30 cycles (94°C × 1 min, 55°C × 1 min, 72°C × 1 min). The PCR products were digested with *Sfi*I and gel purified. A positive control phage displaying the last exon of the IL-4 cDNA (490–612 bp) was also constructed [26].

A phage display vector, pORF-1, was engineered for gene fragment phage display. It is a pHEN-1 [12] based M13-filamentous phagemid vector that contains a *pelB* leader sequence, an upstream hexahistidine tag and a non-religatable *Sfi*I insert cloning site which is contiguous with a myc epitope tag and M13 gene III. pORF-1 was constructed by two rounds of template mutagenesis of pHEN-1 vector with primers (NSFI 5′-GCGGCCCAGCCGGC GATGGCCCAGCACCATCACCATCATCACGG GGCCATGGTGCAGCTGCAGG; SUP 5′-TCA CGGGGCCATGGGGGCCCAGGCCTCAGTCG ATCGACACGGCCTCCACGGCCGCAGAACAA) [16]. The base vector contained an out-of-frame

Copyright © 2002 John Wiley & Sons, Ltd. 

1 kb stuffer fragment. *Sfi*I digested insert was ligated into the digested vector and optimized ligation products were electroporated into *E. coli* TG-1. The size distribution of library inserts was evaluated by PCR with primers flanking the cloning site (*Sfi*seq5 5′-TCACCATCATCACGGGGCCAT, *Sfi*seq3 5′-GTTTTTGTTCTGCGGCCGTTG) with *Pfu* Polymerase for 30 cycles (94°C × 1 min, 55°C × 1 min, 72°C × 1 min).

### Selection and screening of H11 epitope library

Antibodies specific for human IL-4 (C19; Santa Cruz Biotechnology, Santa Cruz, CA) (Mab604; R&D Systems, Minneapolis, MN) and IL-13 (IL13C; Santa Cruz Biotechnology) were obtained from commercial sources. Epitope selections were performed as previously described [19,23], using $10^{12}$ K07-rescued phage particles/ml and (50 μg/ml) antibody-coated immunotubes (Nunc). Random clones from the second round of selection were screened by phage-ELISA on microtiter plates (Corning) coated overnight at 4°C with 25 μg/ml of antibody. Binding of phage was detected with 1 : 1000 horseradish peroxidase-conjugated anti-M13 (Amersham Pharmacia, Piscataway, NJ). Phage displaying epitopes did not cross-react with plastic, albumin, or IgG as determined by ELISA. Positive controls included an IL-4 phage. Insert size of ELISA positive clones was determined by PCR and clones with unique insert size were DNA sequenced and aligned by BLAST. Selections were repeated in cases where no enrichment occurred.

### Determination of epitope clone specificity

The specificity of phage epitope clones for the human IL-4 epitope was determined by competition ELISA using a specific blocking peptide, SC-1260 (Santa Cruz Biotechnology), corresponding to the epitope for the anti-IL-4 antibody C19. ELISA was performed as described above, except that the C19 antibody was preincubated with increasing concentrations (0 to 20 mg/ml) of SC-1260 prior to incubation with phage epitopes. A phage displaying coverage of the last exon of the IL-4 cDNA served as positive control.

### cDNA epitope library construction and selection

A cDNA phage display library was constructed from a breast cancer SKBR-3 cDNA library (Origene, Rockville, Maryland). This oligo-dT-primed library required modification prior to cloning into the phage display vector. 10 μg of cesium chloride purified library plasmid was linearized at the 3′ insert cloning site with *Xba*I. A series of timed nested upstream 3′ deletions with one unit of *Bal*-31 nuclease (New England Bio Lab) were performed at 30°C for 1, 2, 4, 6, 8, and 10 minutes and pooled. DNA was blunted with 5units/μg T4 polymerase for 30 min at 12°C, extracted and precipitated. Linkers containing a *Sfi*I cloning site (Link1 5′-AGCGGCCG CAGGCCATGGAGGCC, Link2 5′-GGCCTCCA TGGCCTGCGGCCGCT) were ligated to the 3′ region with 10units T4 ligase for 15 minutes at room temperature. Template for insert cloning was generated by high stringency PCR with primers to introduce an additional 5′-*Sfi*I cloning site (new_XBAI_for 5′-GCTCTAGAGGACAAACCACAA CTAGAATGCAGTG, LP5 5′-AGCGGCCGCAG GCCATGGA) with *Pfu* Polymerase for 30 cycles (94°C × 1 min, 55°C × 1 min, 72°C × 1.5 min). 10 μg of template PCR product was digested with *Sfi*I and ligated into the pORF-1 vector. Optimized ligation products were electroporated into *E. coli* TG-1. Library characterization was performed in similar fashion as the H11 library by determining insert size and DNA sequence of random clones. The percent of functional display of the library was assessed by detection of the C-terminal myc epitope tag of the phage pIII-fusion proteins. Phage were prepared from random clones from the unselected cDNA epitope library and were screened by phage-ELISA on microtiter plates (Corning) coated with 25 μg/ml 9E10, anti-myc antibody [8]. Binding of phage was detected with 1 : 1000 horseradish peroxidase-conjugated anti-M13 (Amersham Pharmacia). cDNA epitope phage were prepared and selected using immunotubes coated with 50 μg/ml Herceptin® (Trastuzumab, 4D5, Genentech, South San Francisco, CA). Herceptin® reactivity was confirmed by dot blot against purified c-erbB2 extracellular domain.

### Results

### H11 genomic epitope display library

An epitope phage display library, optimized to contain exon-sized inserts, was generated from a 50 kb P1/BAC clone that contained the human Interleukin-4, Interleukin-13, and kinesin-like protein-3

Copyright © 2002 John Wiley & Sons, Ltd. 

genes. The genomic DNA was randomly digested using DNase I. Fragments approximating 100–300 bp were isolated by gel electrophoresis and cloned into the *Sfi*I site of the pORF-1 phagemid vector. The fragment sizes were selected to maximize enrichment of exons (Figure 1). Selection of the target insert size range to maximize exon display was based upon *in silico* analyses of the size distribution of exons in genes within the H11 P1 (Figure 1). Long fragments (>300 bp) are more likely to contain intron sequence with stop codons, which would prevent translation of displayed protein (Figure 1), thereby reducing the diversity and complexity of the library. However, short fragments have a lower likelihood of folding into a domain structure, which could mimic the conformational epitopes that antibodies typically recognize. Thus, while longer fragments are better for domain structure, introns and stop codons pose potential problems in these longer fragments. Estimates of the frequency of stop codons occurring in random DNA or human intron sequence, suggest that 90% of fragments of 150 bp or longer will contain a stop codon. Figure 1 (shaded area) indicates that 90% of the open reading frames from the H11-5q31 region are <200 bp. Thus if a short exon (e.g. 50 bp) is contained within a fragment of >150 bp, there is a high probability that the sequence contains a stop codon and thus cannot be displayed on the surface of the phage. Thus an empirical compromise suggests that libraries from 100–300 bp are optimal to select genomic fragments without stop codons. The size distribution of fifteen random, unselected clones from the genomic library was determined using PCR. The majority of clones (12/15) contained an average insert size of 150 bp with a range of 80–300 bp (Figure 2). DNA sequencing of random clones revealed fragments of genomic

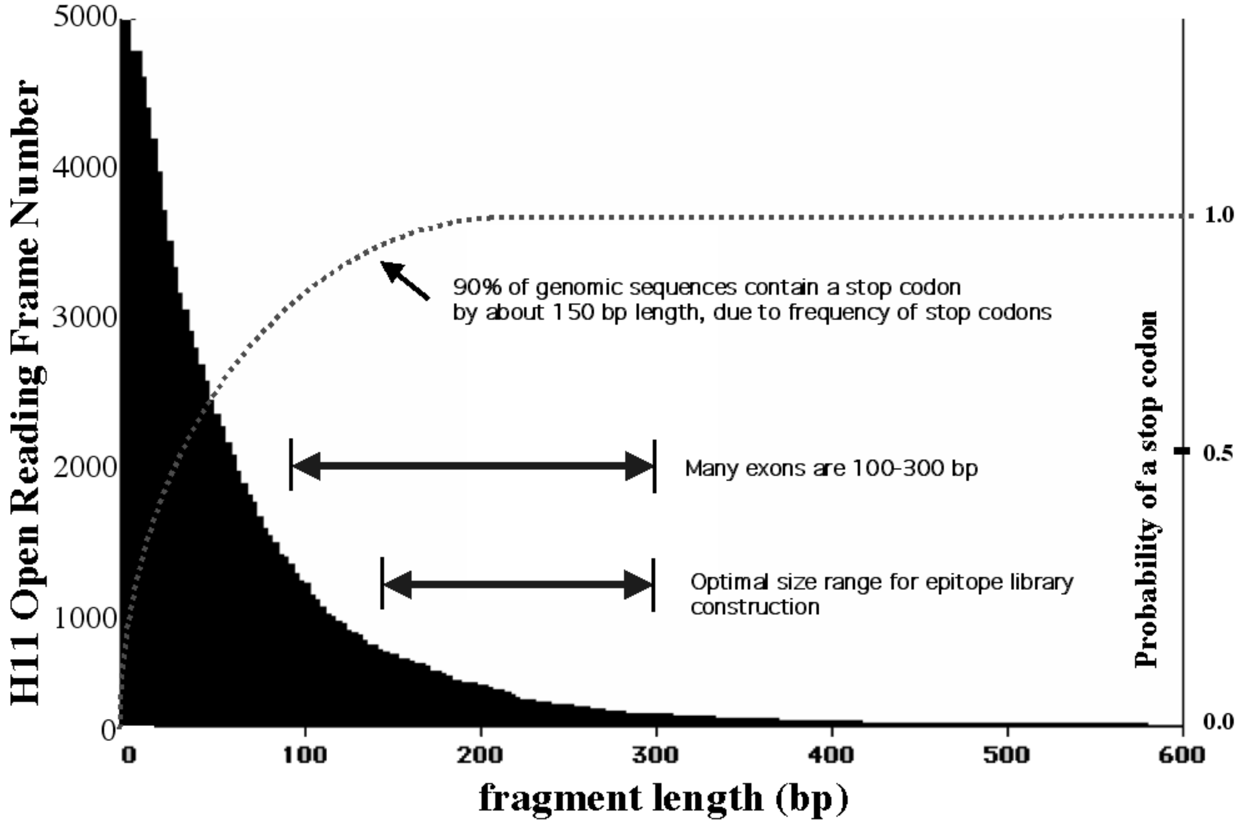

**Figure 1.** Theoretical considerations for genomic epitope display of 5q31. All open reading frames from the 50 kb P1H11 were calculated and compared to exon size of 5q31 genes. The majority of exons in H11 ranged between 100-300 bp. The number of open reading frames (left ordinate) and the probability of a stop codon (right ordinate) were calculated and are displayed as a function of fragment length (absissca)

Copyright © 2002 John Wiley & Sons, Ltd. 

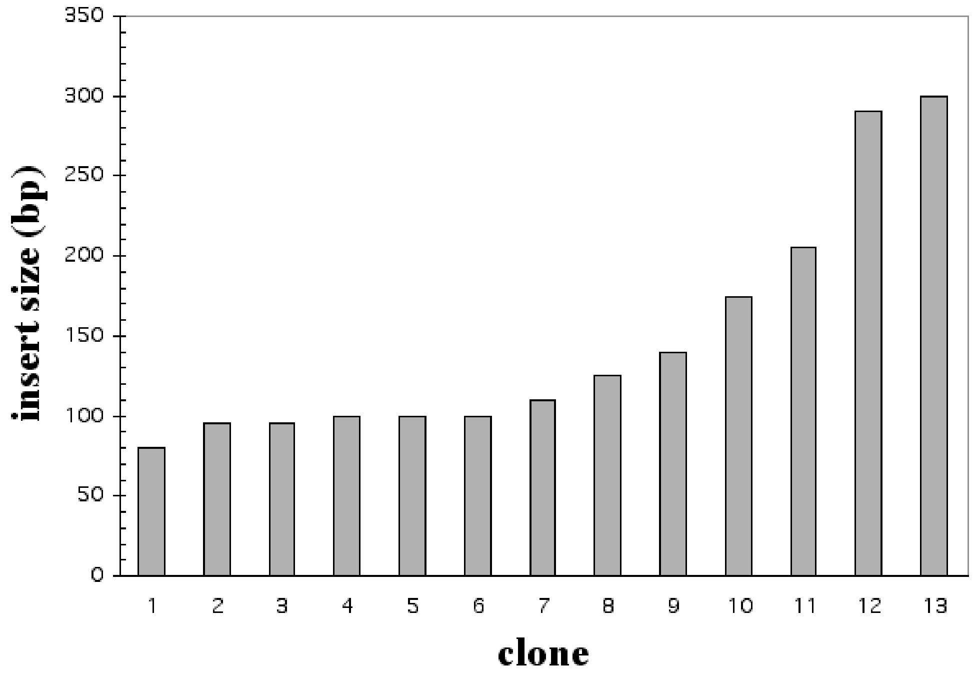

**Figure 2.** Size distribution of genomic inserts from the unselected H11 epitope phage library. Cloned insert DNA was amplified from 13 individual random clones using PCR and primers that flank the insert cloning site and analyzed on a 2.0% agarose gel sequence in both coding orientations. Approximately 5/13 random clones contained DNA sequence that corresponded to *E. coli* genomic sequence and 8/13 clones contain human intron genomic sequence. The library of $2 \times 10^6$ clones appeared to be sufficiently large to cover the sequence space anticipated for a 50–100 kb BAC library ($>>10^5$ clones) and clones contained human intron fragment sizes in the desired exon-size range.

### Antibody selection of H11 genomic library members

Enrichment of exon-based epitope sequences, corresponding to genes within the 5q31/H11 locus, was demonstrated by selecting the genomic epitope library using antibodies specific for the proteins encoded by 5q31/H11 exons. Monoclonal (Mab604) and polyclonal (C19) antibodies against Interleukin-4 or Interleukin-13 (IL13C) were used for epitope selection (Table 1). The C19 antibody was raised against the C-terminal peptide of IL-4 and corresponds to exon 4 of IL-4 [18]. Significant enrichment of the H11 library occurred after two rounds of selection against all three antibodies, as indicated by increasing phage titers (1–3 orders of magnitude per selection round) (Table 1). More than 50% of individual clones screened by phage-ELISA were positive after the second round of selection. DNA sequencing revealed unique clones against each antibody. Most clones contained similar sized inserts (Table 1). The DNA sequence of fifteen positive clones was determined. Two unique clones were identified using C19 anti-IL-4 antibody selection. One clone (H11_207) matches the human Interleukin-4 epitope: consisting of an IL-4 fusion product composed of a 46 bp human telomeric sequence (2PTEL066, 176–130 bp) and the IL4 cDNA sequence from exon 4 (AC004039, 24244–24170 bp). Unexpectedly, other clone insert sequences corresponded to *E. coli* enomic DNA. 8/9 clones (clone H11_201) matched a 54 bp insert sequence from *E. coli* heR/CheB chemotaxis/transferase genes (ECOCHE3, 28–71 bp, accession #M13463). The Mab604 anti-IL4 antibody selection resulted in isolation of two unique clones of 800 bp corresponding to two distinct contaminating human single-chain antibody sequence. The IL13C selection resulted in

Table 1. Phage selection and enrichment of the H11 library during selection rounds. The output titer of phage recovered after each round of selection was determined. Individual clones from the second round selection were isolated, phage prepared, and analyzed by ELISA for binding to C19, Mab604, and IL13C antibodies. Binding clones were analyzed by PCR to determine insert size and DNA sequencing

| Antibody | C19 | Mab604 | IL13C |
|---|---|---|---|
| target | anti-IL-4 | anti-IL-4 | anti-IL-13 |
| immunogen | C-terminal peptide | recombinant protein | C-terminal peptide |
| clonality | polyclonal | monoclonal | polyclonal |
| purification | Serum | Protein G purified | Protein G purified |
| Round 1 Input Titer (cfu) | $1 \times 10^{12}$ | $1 \times 10^{12}$ | $1 \times 10^{12}$ |
| Round 1 Output Titer (cfu) | $3 \times 10^{3}$ | $1 \times 10^{5}$ | $2 \times 10^{4}$ |
| Round 2 Input Titer (cfu) | $1 \times 10^{12}$ | $1 \times 10^{12}$ | $1 \times 10^{12}$ |
| Round 2 Output Titer (cfu) | $6 \times 10^{5}$ | $3 \times 10^{7}$ | $5 \times 10^{7}$ |
| Round 2 ELISA positive clones | 60/96 | 46/96 | 77/96 |
| Unique clones by PCR size | 2/30 | 2/20 | 4/20 |
| Unique clones by DNA sequence | 2/9 | 2/2 | 1/4 |

isolation of four identical clones of 110 bp that matched *E. coli* ep helicase (ECREPHEL, 329–219 bp, accession #X04794) sequence. The specificity of phage clones for the human IL-4 epitope was demonstrated by competition ELISA using the specific C19 blocking peptide, SC-1260. Binding of both the IL-4 epitope (clone H11_207) and the IL-4 mimotope (clone H11_201) to antibody was displaced with increasing concentrations of peptide, confirming the IL-4 specificity of the phage epitopes (Figure 3).

### SKBR-3 cDNA display library

After establishing that the epiotope display library contained antibody-binding mimitopes, we pursued a cDNA phage library approach. A cDNA phage display library was constructed from the SKBR3 breast cancer cell line. The parental cDNA library was oligo-dT primed (i.e. contains a 3′ stop codon), and thus required 3′ modification to prevent a stop codon within the pIII-fusion. The phage library contained $3 \times 10^7$ clones with insert size ranging between 200–1000 bp, corresponding to domain-sized fragments (Figure 4). Preliminary library characterization using the myc epitope tag indicated functional display of $> 10^6$ clones (11/95 randomly selected clones were ELISA positive). DNA sequencing of random clones from the unselected library revealed only human sequences (400 bp fragment corresponding to human S19 ribosomal protein; 250 bp human ribosomal protein RPL13/S11, U32-UU35; 350 bp homology to drosophila BcDNA GM013838; 240 bp human BAC G1-214N3; 500 bp human 80 kd heat shock protein; 650 bp human KIAA0466 protein). Bacterial sequences were not detected in the SKBR3 display library. Phage epitopes were selected using the Herceptin® monoclonal antibody, which binds c-erbB2, a cell surface receptor over-expressed in the SKBR3 cell line. Enrichment of antibody binders occurred after two selection rounds: the titers of recovered phage increased between round 1 ($10^5$ cfu) and round 2 ($10^6$ cfu). After two selection rounds, 6/95 phage clones bound Herceptin by ELISA. Enrichment decreased with a third round of selection, despite repeated selections (Round 3, $10^5$ cfu). Six clones from Round 2 were sequenced; however, none matched a sequence corresponding to human c-erbB2 (Table 2). Rather, the clones contained human sequences that matched enzymes, signaling molecules, and structural proteins (Table 2).

### Discussion

The ability to identify coding sequences within a genomic interval using a proteomic approach is important to complement current genome-protein strategies that rely solely upon DNA sequence information. Coupling epitope display libraries from human genomic regions with antibody selection for expressed epitopes has the potential to enrich for library members with coding (exon) sequences. A phage display library containing the full human genome sequence could be an invaluable tool for human protein-protein interaction identification. Some variables to consider in construction of such

Copyright © 2002 John Wiley & Sons, Ltd. 

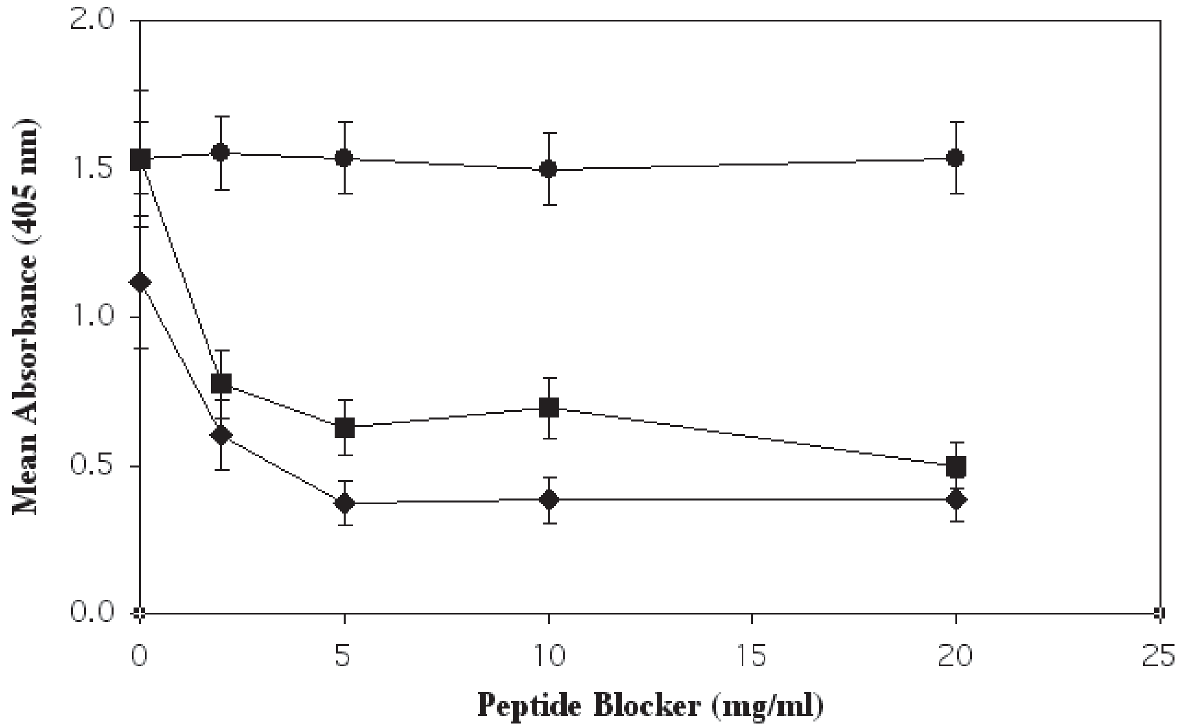

**Figure 3.** Specificity of mimotope clones for IL-4 by competition ELISA. The anti-IL-4 antibody C19 was preincubated with or without increasing concentrations (0–20 mg/ml) of specific blocking peptide SC-1260 prior to ELISA with phage epitope (H11_207) and mimotope (H11_201) clones. (H11_201 without peptide, circle; H11_201 with peptide, square; H11_207 with peptide, diamond). Data are representative of two experiments a complete library include the DNA sequence space to be covered ($3 \times 10^9$ bp human genome), the insert size (e.g., 100–300 bp exon size), number of partial overlapping inserts desired (diversity), and functional peptide display due to stop codon effects on display. For example, the absolute minimum number of members in a library of the human genome composed of 150 bp inserts would be about $2 \times 10^7$ members ($3 \times 10^9$ bp/150 bp); however, 90% of these would not display peptides due to stop codons in genomic sequence. Thus, it's desirable to generate a library of at least two magnitudes greater size ($>10^9$ members). A library with large diversity favors potential binders; however, large libraries are difficult to construct. A practical limitation is the efficiency of transforming bacteria with plasmid,

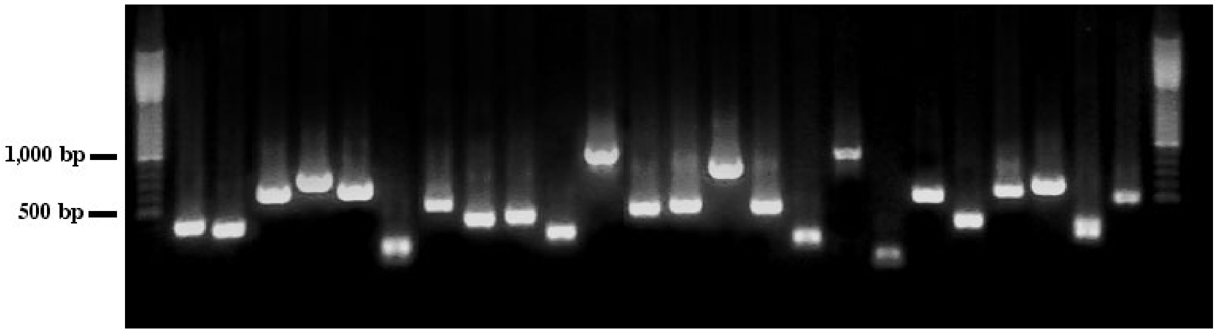

**Figure 4.** Size distribution of PCR inserts from the unselected SKBR-3 cDNA phage library. Cloned insert DNA was amplified from individual random clones using PCR and primers that flank the insert cloning site and analyzed on a 1.0% agarose gel

Copyright © 2002 John Wiley & Sons, Ltd. 

Table 2. Phage selection of SKBR-3 mimotope cDNA clones against Herceptin® monoclonal antibody. ELISA positive clones from the second round of selection were DNA sequenced and the corresponding genes identified

| Clone ID | Database ID | Database Match | Start–end (bp) |
|---|---|---|---|
| SKH201 | HSM801448 | human cDNA DKFZp434E012 | 1310–1740 |
| SKH202 | S7547 | human phosphoglycerate kinase 1 | 15–155 |
| SKH203 | AC016722 | human clone RP11-333I13 | 132054–904 |
| SKH204 | XM_034498.1 | human farnesyl diphosphate synthetase protein | 873–1022 |
| SKH205 | AK021828 | human KIAA1293 | 878–1014 |
| SKH206 | AY012136.1 | human mitochondrial 12S rRNA | 17–309 |

typically about $10^8$–$10^9$ colony-forming units per microgram of DNA. Nonetheless, construction of large, functional, genome display libraries is feasible as recently demonstrated with the *Toxoplasma gondii* genome [21].

To determine the utility of using a human genomic library approach to select coding regions, an epitope phage display library was constructed using exon-sized fragments from a human genomic region containing the 5q31 Interleukin gene cluster. The size-selected library contained sufficient diversity to cover the sequence space for a 50 kb BAC with 100 bp inserts. Selection of the epitope library using anti-Interleukin antibodies enriched for phage members containing 'true' Interleukin epitopes, as well as for other sequences. An IL-4 epitope sequence was selected from the library against the C19 antibody, which recognizes the C-terminal epitope of the last exon of IL-4 [18]. An associated 46 bp unrelated small fragment of human telomeric sequence (2PTEL066, 176-130) was fused to the IL-4 gene and may have resulted from a PCR induced artifact. Detection of the IL-4 epitope sequence demonstrates the feasibility of this exon-based phage display strategy to select peptides that correspond to proteins encoded by sequence in a specified genomic region.

Unexpectedly, other antibody binding phage clones contained sequences that correspond to *E. coli* genomic DNA. The bacterial sequences encode peptides that specifically bind to the anti-IL-4 antibody and this binding can be blocked with the 'true' peptide epitope. Thus, the peptides encoded by bacterial genome sequence act as 'mimotopes' (mimetic sequences of the true epitope). These mimotopes correspond to open reading frames, which generate displayed peptides that compete for binding during phage selection. The CheR/CheB sequence (clone H11_201) is a specific IL-4 mimotope as confirmed by competition ELISA (Figure 3). Computational alignment analysis indicated that the IL-4 mimotope lacked homology with the IL-4 epitope. It is likely that the presence of *E. coli* DNA during the preparation of P1's and BAC's contribute to selection of these mimotopes. This epitope library contained approximately 40% (5/13) bacterial sequence. *E. coli* contamination may be particularly problematic during purification because these human artificial chromosomes are present at low copy number. It is possible that *E. coli* sequences are preferentially amplified during PCR due to low specificity primers in the presence of greater abundance of bacterial DNA. *E. coli* sequence in the libraries persisted, even with the use of rigorous protocols with ultrapure reagents. Although further rounds of selection might be anticipated to identify a human epitope binder, studies with our previous epitope libraries for Botulinum toxin [19] indicated that greater than two-three rounds of selection rarely improved discrimination of true binders from mimotopes. Furthermore, other *E. coli* mimotopes were generated against other antibodies: C25 mAb (mimotope database accession numbers: L10328, U70214, AE000354, L02370) and S25 mAb (mimotope database accession numbers: X58994, X51655, AE000177) [19].

Genome-protein linkage using a phage display two-hybrid approach to detect epitopes encoded by specified genomic regions requires effective binding of displayed peptides and identification of true binders versus mimotopes. Library members containing bacterial DNA sequence can easily be excluded simply from further analysis by DNA sequencing; however, false positives generated by these mimotopes decrease the overall screening efficiency. Furthermore, since phage display selection is based on affinity interactions, mimotopes that are more effectively expressed than true epitopes and/or have

Copyright © 2002 John Wiley & Sons, Ltd. 

higher affinities may compete more effectively than lower affinity epitopes. Similar cross-reactive epitope mimics within a fragment display library of the *Rickettsia* genome have been described by Fehrsen *et al.* [10]. In our studies, the C19 and IL13C phage selections were performed with commercially available polyclonal antisera generated using IL-4/IL-13 peptide immunogens. Adjuvants, frequently used in peptide-based immunizations, commonly contain bacterial proteins. Thus, polyclonal antisera that contain IgGs against target proteins (IL-4/IL-13) may also contain a background of IgGs against many *E. coli* proteins. Mimotopes are not problematic in a single chain antibody library because all binders are mimetics, and thus are considered to be positive binders, regardless of whether they are true epitopes or mimotopes. However, our studies suggest that enrichment of background bacterial epitopes may be a common feature and limitation of genomic phage display based selection/screening strategies. Identification of true epitopes is critical for genome-protein linkage.

We hypothesized that potential mimotope problems may be avoided using cDNA phage libraries because cDNA covers the protein coding sequence space more efficiently than genomic libraries. The advantages of cDNA libraries include the absence of intron sequence, expression of higher copy plasmid compared to artificial chromosomes, and potentially less *E. coli* interference. To test this concept, we used a breast cancer SKBR-3 cDNA phage display library that contained at least $10^6$ displayed clones with appropriate domain size sequences. We anticipated that we could enrich breast cancer specific sequences, such as the highly abundant c-erbB2 cDNA. Thus, we selected this library against Herceptin® a humanized murine monoclonal antibody directed against the extracellular domain of c-erbB2. Although multiple selection rounds indicated enrichment, only non-erbB2, mimotope sequences were identified (Table 2). PCR analysis indicated that the erbB2/Herceptin® epitope sequence is present within the phage library (data not shown); however, the frequency of clones expressing peptides derived from this sequence cannot be established because we were unable to isolate this clone. Large scale screening of additional clones may be necessary to identify binders for the short linear sequence of c-erbB2 near the transmembrane region, recognized by Herceptin®. Alternatively, the c-erbB2 epitope may be poorly expressed or displayed on phage, although we were able to select an c-erbB2 epitope from a phage display epitope library constructed by fragmenting the c-erbB2 cDNA (data not shown).

Successful demonstration of the phage two-hybrid protein-protein interaction approach to identify expressed proteins from specified genomic regions is encouraging. However, an effective two-hybrid phage display system will require optimization of numerous variables including conformational versus linear epitopes, high versus low affinity interactions, coding versus non-coding sequence space, and competition from irrelevant background sequences. The problem of human and bacterial mimotopes may be reduced if monoclonal antibodies or single chain phage antibodies are used instead of polyclonal serum. The phage two-hybrid approach is limited to continuous epitopes, which meet the size constraint criteria. Thus, conformational (discontinuous) epitopes or those that exceed the average size of a single exon will not be detected. Exons within 5q31 represent $<1\%$ of the genomic sequence coding for proteins in the P1 clone. In the absence of a 'true' epitope within the library, irrelevant background binders have a better chance to compete and bind the target antibody. The promiscuity of phage displayed sequences, such as unexpected frame shifts or sequences containing obvious stop codons [4,11], are also important considerations for phage based antibody-antigen interactions. False-positives are common features of most other two-hybrid approaches. For example, in a recent study cDNA T7 phage display linked the kinase domain of the EGF receptor to the Ras/MAP kinase pathway [27]; however, a variety of other binders (i.e., false positives) are listed. Our data indicate that protein mimotopes will complicate most genome-proteome approaches that require detection of highly specific and cognate protein-protein interactions for functional genomics and proteome validation.

Although we describe the complications of mimotopes in a two-hybrid phage display approach, we also show that a library containing *E. coli* mimotopes is a rich source of peptide binders against target antigens. The *E. coli* genome contains a higher proportion of coding to non-coding sequence than the human genome; thus, it may represent a useful DNA sequence source to generate peptide diversity biased towards protein coding sequences. We speculate that *E. coli* mimotopes may be more antigenic than the cognate human peptides. Additional studies are warranted to explore this possibility, since bacterial mimotopes of human peptides might

Copyright © 2002 John Wiley & Sons, Ltd. 

be useful for applications such as vaccine development.

### Acknowledgement

We thank Dr. Lewis Lanier (University of California at San Francisco) for generously providing the human IL-4 cDNA and Dr. Edward Reuben (Lawrence Berkeley National Laboratory) for the H11 P1.

### References

1. Burge C, Karlin S. 1997. Prediction of complete gene structures in human genomic DNA. *JMol Biol* **268**: 78–94.
2. Burton DR. 1995. Phage display. *Immunotech* **1**: 87–94.
3. Cabilly S. 1999. The basic structure of filamentous phage and its use in the display of combinatorial peptide libraries. *Mol Biotech* **12**: 143–148.
4. Carcamo J, Ravera MW, Brissette R, *et al.* 1998. Unexpected frameshifts from gene to expressed protein in a phage-displayed peptide library. *Proc Natl Acad Sci U S A* **95**: 11146–11151.
5. Cochrane D, Webster C, Masih G, *et al.* 2000. Identification of natural ligands for SH2 domains from a phage display cDNA library. *J Mol Biol* **297**: 89–97.
6. Collins C, Rommens JM, Kowbel D, *et al.* 1998. Positional cloning of ZNF217 and NABC1: genes amplified at 20q13.2 and overexpressed in breast carcinoma. *Proc Natl Acad Sci U S A* **95**: 8703–8708.
7. Cortese R, Monaci P, Luzzago A, *et al.* 1996. Selection of biologically active peptides by phage display of random peptide libraries. *Curr Opin Biotech* **7**: 616–621.
8. Evan GI, Lewis GK, Ramsay G, *et al.* 1985. Isolation of monoclonal antibodies specific for human c-myc proto-oncogene product. *Mol Cell Biol* **5**: 3610–3616.
9. Fack F, Hugle-Dorr B, Song D, *et al.* 1997. Epitope mapping by phage display: random versus gene-fragment libraries. *J Immun Meth* **206**: 43–52.
10. Fehrsen J, du Plessis DH. 1999. Cross-reactive epitope mimics in a fragmented-genome phage display library derived from the rickettsia, *Cowdria ruminantium*. *Immunotech* **4**: 175–184.
11. Goldman E, Korus M, Mandecki W. 2000. Efficiencies of translation in three reading frames of unusual non-ORF sequences isolated from phage display. *Faseb J* **14**: 603–611.
12. Hoogenboom HR, Griffiths AD, Johnson KS, *et al.* 1991. Multi-subunit proteins on the surface of filamentous phage: methodologies for displaying antibody (Fab) heavy and light chains. *Nuc Acids Res* **19**: 4133–4137.
13. Hufton SE, Moerkerk PT, Meulemans EV, *et al.* 1999. Phage display of cDNA repertoires: the pVI display system and its applications for the selection of immunogenic ligands. *J Immuno Meth* **231**: 39–51.
14. Jacobsson K, Frykberg L. 1995. Cloning of ligand-binding domains of bacterial receptors by phage display. *Biotechniques* **18**: 878–885.
15. Jacobsson K, Frykberg L. 1996. Phage display shot-gun cloning of ligand-binding domains of prokaryotic receptors approaches 100% correct clones. *Biotechniques* **20**: 1070–1091.
16. Kunkel TA, SoniA. 1988. Mutagenesis by transient misalignment. *J Biol Chem* **263**: 14784–14789.
17. Marks JD, Hoogenboom HR, Bonnert TP, *et al.* 1991. Bypassing immunization. Human antibodies from V-gene libraries displayed on phage. *J Mol Biol* **222**: 581–597.
18. Mullaney BP, Pallavicini MG. 2001. Protein–protein interactions in hematology and phage display. *Exp Hem* **29**: 1136–1146.
19. Mullaney BP, Pallavicini MG, Marks JD. 2001. Epitope mapping of neutralizing Botulinum A antibodies by phage display. *Infection Immun* **69**: 6511–6514.
20. Pereboeva LA, Pereboev AV, Wang LF, *et al.* 2000. Hepatitis C epitopes from phage-displayed cDNA libraries and improved diagnosis with a chimeric antigen. *J Med Virol* **60**: 144–151.
21. Robben J, Hertveldt K, Bosmans E, *et al.* 2002. Selection and identification of dense granule antigen GRA3 by *Toxoplasma gondii* whole genome phage display. *J Biol Chem* (in press).
22. Santi E, Capone S, Mennuni C, *et al.* 2000. Bacteriophage lambda display of complex cDNA libraries: a new approach to functional genomics. *J Mol Biol* **296**: 497–508.
23. Schier R, McCall A, Adams GP, *et al.* 1996. Isolation of picomolar affinity anti-c-erbB-2 single-chain Fv by molecular evolution of the complementarity determining regions in the center of the antibody binding site. *J Mol Biol* **263**: 551–567.
24. Scott JK, Smith GP. 1990. Searching for peptide ligands with an epitope library. *Science* **249**: 386–390.
25. Xu Y, Mural R, Shah M, *et al.* 1994. Recognizing exons in genomic sequence using GRAIL II. *Genetic Eng* **16**: 241–253.
26. Yokota T, Otsuka T, Mosmann T, *et al.* 1986. Isolation and characterization of a human interleukin cDNA clone, homologous to mouse B-cell stimulatory factor 1, that expresses B-cell- and T-cell-stimulating activities. *Proc Natl Acad Sci U S A* **83**: 5894–5898.
27. Zozulya S, Lioubin M, Hill RJ, *et al.* 1999. Mapping signal transduction pathways by phage display. *Nat Biotech* **17**: 1193–1198.

Copyright © 2002 John Wiley & Sons, Ltd. 

## References

[PDF_00700] Burge C., Karlin S. (1997). Prediction of complete gene structures in human genomic DNA.. J Mol Biol.

[PDF_00702] Burton D. R. (1995). Phage display.. Immunotechnology.

[PDF_00703] Cabilly S. (1999). The basic structure of filamentous phage and its use in the display of combinatorial peptide libraries.. Mol Biotechnol.

[PDF_00710] Cochrane D., Webster C., Masih G., McCafferty J. (2000). Identification of natural ligands for SH2 domains from a phage display cDNA library.. J Mol Biol.

[PDF_00713] Collins C., Rommens J. M., Kowbel D., Godfrey T., Tanner M., Hwang S. I., Polikoff D., Nonet G., Cochran J., Myambo K. (1998). Positional cloning of ZNF217 and NABC1: genes amplified at 20q13.2 and overexpressed in breast carcinoma.. Proc Natl Acad Sci U S A.

[PDF_00717] Cortese R., Monaci P., Luzzago A., Santini C., Bartoli F., Cortese I., Fortugno P., Galfrè G., Nicosia A., Felici F. (1996). Selection of biologically active peptides by phage display of random peptide libraries.. Curr Opin Biotechnol.

[PDF_00706] Cárcamo J., Ravera M. W., Brissette R., Dedova O., Beasley J. R., Alam-Moghé A., Wan C., Blume A., Mandecki W. (1998). Unexpected frameshifts from gene to expressed protein in a phage-displayed peptide library.. Proc Natl Acad Sci U S A.

[PDF_00720] Evan G. I., Lewis G. K., Ramsay G., Bishop J. M. (1985). Isolation of monoclonal antibodies specific for human c-myc proto-oncogene product.. Mol Cell Biol.

[PDF_00723] Fack F., Hügle-Dörr B., Song D., Queitsch I., Petersen G., Bautz E. K. (1997). Epitope mapping by phage display: random versus gene-fragment libraries.. J Immunol Methods.

[PDF_00726] Fehrsen J., du Plessis D. H. (1999). Cross-reactive epitope mimics in a fragmented-genome phage display library derived from the rickettsia, Cowdria ruminantium.. Immunotechnology.

[PDF_00730] Goldman E., Korus M., Mandecki W. (2000). Efficiencies of translation in three reading frames of unusual non-ORF sequences isolated from phage display.. FASEB J.

[PDF_00733] Hoogenboom H. R., Griffiths A. D., Johnson K. S., Chiswell D. J., Hudson P., Winter G. (1991). Multi-subunit proteins on the surface of filamentous phage: methodologies for displaying antibody (Fab) heavy and light chains.. Nucleic Acids Res.

[PDF_00737] Hufton S. E., Moerkerk P. T., Meulemans E. V., de Bruïne A., Arends J. W., Hoogenboom H. R. (1999). Phage display of cDNA repertoires: the pVI display system and its applications for the selection of immunogenic ligands.. J Immunol Methods.

[PDF_00741] Jacobsson K., Frykberg L. (1995). Cloning of ligand-binding domains of bacterial receptors by phage display.. Biotechniques.

[PDF_00744] Jacobsson K., Frykberg L. (1996). Phage display shot-gun cloning of ligand-binding domains of prokaryotic receptors approaches 100% correct clones.. Biotechniques.

[PDF_00748] Kunkel T. A., Soni A. (1988). Mutagenesis by transient misalignment.. J Biol Chem.

[PDF_00750] Marks J. D., Hoogenboom H. R., Bonnert T. P., McCafferty J., Griffiths A. D., Winter G. (1991). By-passing immunization. Human antibodies from V-gene libraries displayed on phage.. J Mol Biol.

[PDF_00756] Mullaney B. P., Pallavicini M. G., Marks J. D. (2001). Epitope mapping of neutralizing botulinum neurotoxin A antibodies by phage display.. Infect Immun.

[PDF_00753] Mullaney B. P., Pallavicini M. G. (2001). Protein-protein interactions in hematology and phage display.. Exp Hematol.

[PDF_00759] Pereboeva L. A., Pereboev A. V., Wang L. F., Morris G. E. (2000). Hepatitis C epitopes from phage-displayed cDNA libraries and improved diagnosis with a chimeric antigen.. J Med Virol.

[PDF_00767] Santi E., Capone S., Mennuni C., Lahm A., Tramontano A., Luzzago A., Nicosia A. (2000). Bacteriophage lambda display of complex cDNA libraries: a new approach to functional genomics.. J Mol Biol.

[PDF_00770] Schier R., McCall A., Adams G. P., Marshall K. W., Merritt H., Yim M., Crawford R. S., Weiner L. M., Marks C., Marks J. D. (1996). Isolation of picomolar affinity anti-c-erbB-2 single-chain Fv by molecular evolution of the complementarity determining regions in the center of the antibody binding site.. J Mol Biol.

[PDF_00774] Scott J. K., Smith G. P. (1990). Searching for peptide ligands with an epitope library.. Science.

[PDF_00776] Xu Y., Mural R., Shah M., Uberbacher E. (1994). Recognizing exons in genomic sequence using GRAIL II.. Genet Eng (N Y).

[PDF_00778] Yokota T., Otsuka T., Mosmann T., Banchereau J., DeFrance T., Blanchard D., De Vries J. E., Lee F., Arai K. (1986). Isolation and characterization of a human interleukin cDNA clone, homologous to mouse B-cell stimulatory factor 1, that expresses B-cell- and T-cell-stimulating activities.. Proc Natl Acad Sci U S A.

[PDF_00783] Zozulya S., Lioubin M., Hill R. J., Abram C., Gishizky M. L. (1999). Mapping signal transduction pathways by phage display.. Nat Biotechnol.

